# Author Correction: The weather affects air conditioner purchases to fill the energy efficiency gap

**DOI:** 10.1038/s41467-022-33990-7

**Published:** 2022-12-07

**Authors:** Pan He, Pengfei Liu, Yueming (Lucy) Qiu, Lufan Liu

**Affiliations:** 1 School of Earth and Environmental Sciences, Cardiff University, Cardiff, UK; 2 Department of Environmental and Natural Resource Economics, College of the Environment and Life Sciences, University of Rhode Island, Kingston, RI 02881 USA; 3 School of Public Policy, University of Maryland College Park, College Park, MD 20742 USA

**Keywords:** Psychology and behaviour, Environmental economics

**Correction to**: *Nature Communications* 10.1038/s41467-022-33531-2, published online 01 October 2022

The original version of this Article omitted an Acknowledgement of a funding source for the research undertaken, namely “this is a Cardiff EARTH CRediT Contribution 3.” This has been corrected in both the PDF and HTML versions of the Article.

