# Supplementary material for: The weather affects air conditioner purchases to fill the energy efficiency gap
Source: Nat Commun. 2022 Oct 1;13:5772. doi: 10.1038/s41467-022-33531-2 (PMC9526731; doi:10.1038/s41467-022-33531-2)
Supplement: Supplementary file 1 — Supplementary information [file 41467_2022_33531_MOESM1_ESM.pdf]

Supplementary Information

The weather affects air conditioner purchases to fill the energy efficiency gap

Pan He[1], Pengfei Liu[2,*], Yueming (Lucy) Qiu[3,*], Lufan Liu[2]

1. School of Earth and Environmental Sciences, Cardiff University, Cardiff, UK

2. Department of Environmental and Natural Resource Economics, College of the Environment and Life Sciences, University of Rhode Island, Kingston, RI, USA

3. School of Public Policy, University of Maryland College Park, College Park, MD, 20742, USA

* Corresponding authors: Liu pengfei_liu@uri.edu ; Qiu yqiu16@umd.edu

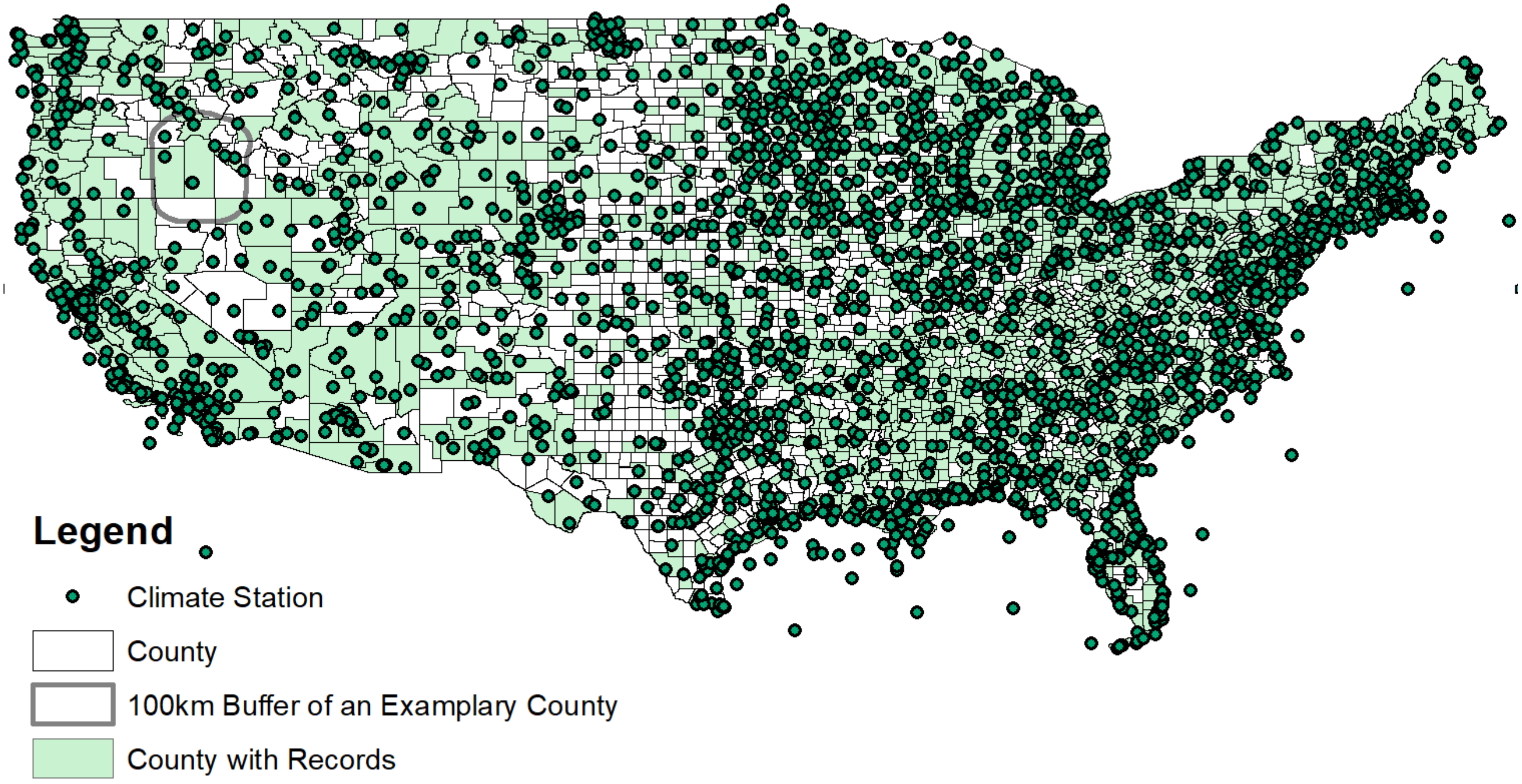

Supplementary Figure 1 Climate stations and counties with records

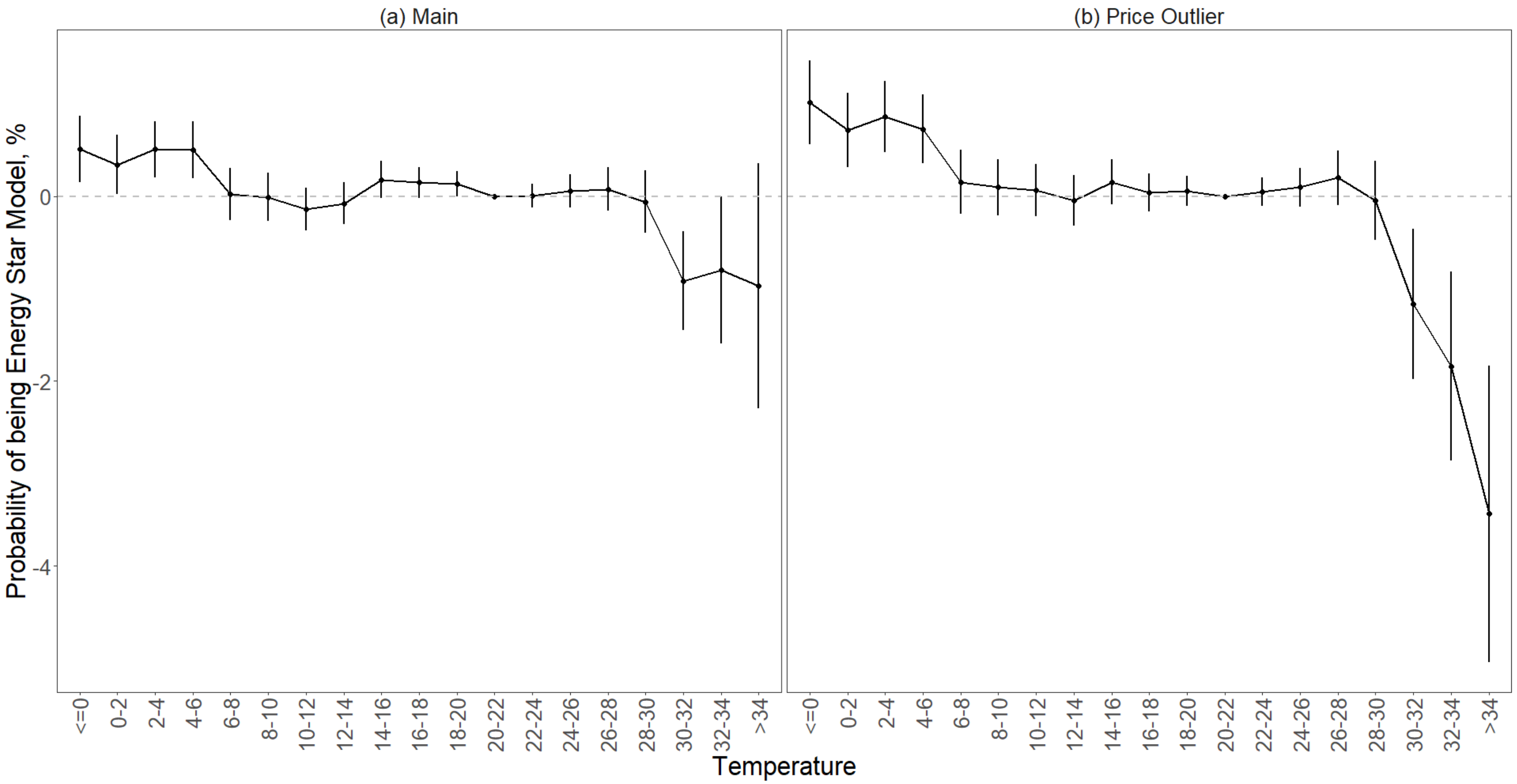

Supplementary Figure 2 The effect of temperature on Energy Star telephone purchase. a) Full sample (N=25395497). b) Records with price<$10 dropped (N=19278726). All the sample sizes refer to transactions from the Nielsen Scanner Dataset. The vertical lines show the 95% confidence intervals (i.e. mean values +/- 2SEM).

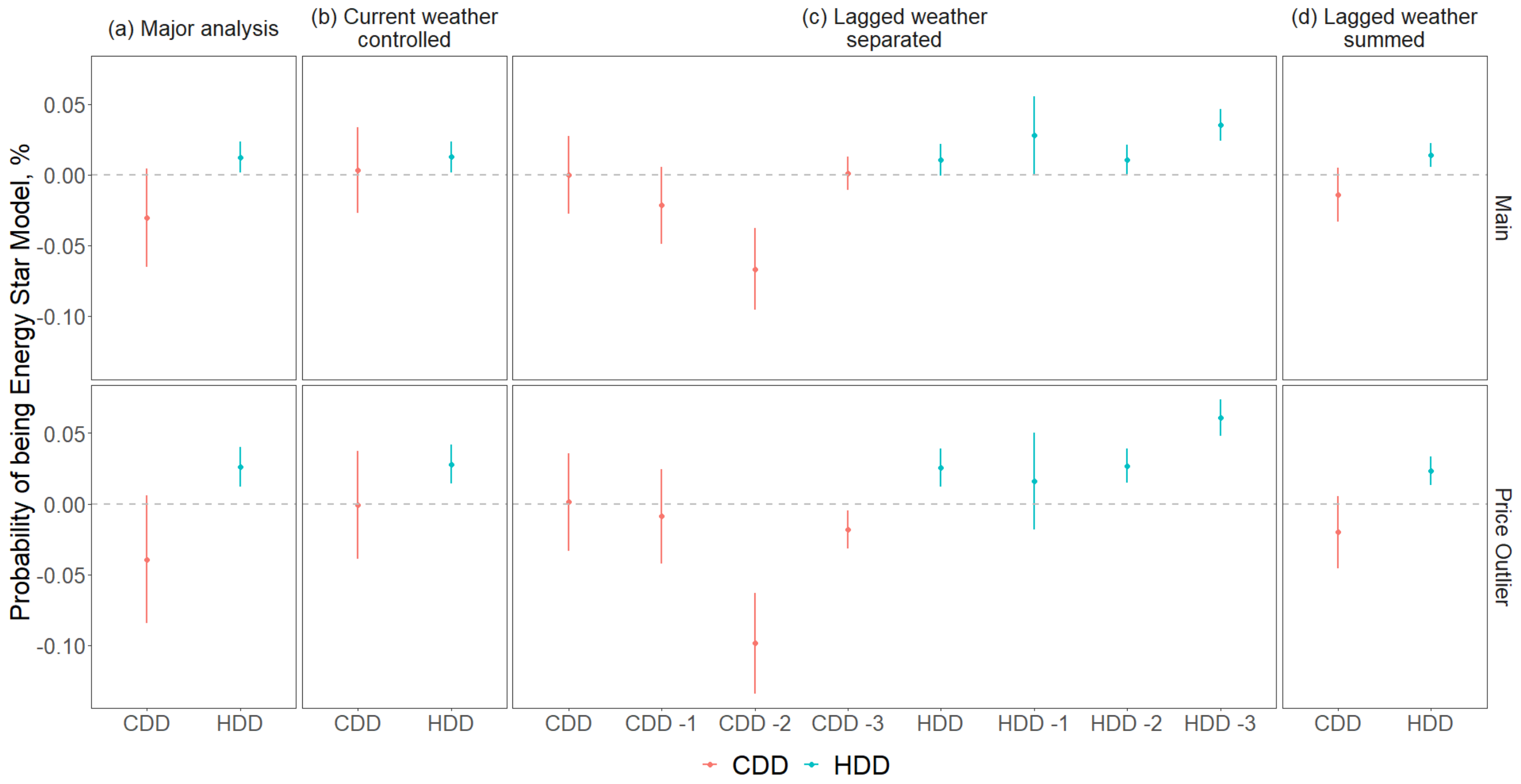

Supplementary Figure 3 The effect of temperature on Energy Star telephone purchase in terms of CDDs and HDDs. a) major analysis using the CDDs and HDDs in the previous week of transaction (N=25395497 for the full sample, 19278726 for the records with price<$10 dropped). b) controlling for the meteorological records in the week of transaction (N=25226932 for the full sample, 19157396 for the records with price<$10 dropped), c) controlling for the meteorological records lagged for 1, 2, and 3 weeks (N=24940700 for the full sample, 18951915 for the records with price<$10 dropped), d) impact of the summed CDDs and HDDs in the previous month of the transaction (N=24940700 for the full sample, 18951915 for the records with price<$10 dropped). All the sample sizes refer to transactions from the Nielsen Scanner Dataset. The vertical lines show the 95% confidence intervals (i.e. mean values +/- 2SEM).

Supplementary Table 1 Descriptive statistics

	N	Mean	SD	Min	Max
Transaction level					
Price ($)	2154198	175.44	87.114	0.01	699.99
Energy Star	1608344	170.196	94.762	0.01	699.99
Non- Energy Star	545854	190.892	56.31	0.01	649.99
County level					
Temperature (°C)	1945092	19.841	5.011	-21.036	40.5
CDDs	1945092	19.816	26.007	0	244.3
HDDs	1945092	35.818	45.042	0	531.05
Wind speed (m/s)	1920585	2.914	0.934	0	21.8
Precipitation (mm)	1944655	2.351	3.067	0	67.157
Relative Humidity (%)	1883615	66.267	11.458	0.3	100
Residential electricity price ($/kwh)	2154197	12.456	2.786	6.88	15.803
CDDs, 1996-2005	2151620	8.753	8.337	0	70.325
HDDs, 1996-2005	2151620	125.552	34.171	9.454	216.324
Median annual household income ($)	1674221	6.70E+04	1.90E+04	2.00E+04	1.40E+05
Population with bachelors above 25 (%)	1674221	0.338	0.109	0.054	0.753
Population of white (%)	1674221	0.733	0.179	0.155	0.998
Median age (Years)	1674221	39.041	3.776	22.7	59.1
Median number of rooms	1674221	5.557	0.696	3.3	7.7
Owner : Renter (Ratio)	1674221	2.04	0.998	0.236	8.599
Electricity as the heating fuel (%)	1674221	0.234	0.197	0.012	0.975
Population believing climate change happening	2151765	66.258	5.807	43.9	80
Population believing climate change will harm the US (%)	2151765	55.176	4.979	41.6	64.8
Population worried about climate change (%)	2151765	55.597	6.694	37.9	70.3
Population supporting renewable energy standards (%)	2151765	57.659	4.387	40.1	66.2
Population supporting regulation of CO_2 as a	2151765	69.246	3.836	49.8	77.9

pollutant (%)					
Support Democratics in election (%)	2151143	0.551	0.158	0.048	0.925

Supplementary Table 2 The effect of weather on Energy Star air conditioner and telephone purchase

	Air conditioner				Telephone	
	(1)	(2)	(3)	(4)	(5)	(6)
	EnergyStar	EnergyStar	EnergyStar	EnergyStar	EnergyStar	EnergyStar
Temperature interval (20-22°C by default)						
<0°C	-0.168***	-0.145***			0.005***	0.010***
	(0.041)	(0.053)			(0.002)	(0.002)
0-2°C	-0.022	-0.008			0.003**	0.007***
	(0.020)	(0.022)			(0.002)	(0.002)
2-4°C	-0.012	-0.000			0.005***	0.009***
	(0.016)	(0.017)			(0.002)	(0.002)
4-6°C	0.014	0.025**			0.005***	0.007***
	(0.011)	(0.012)			(0.002)	(0.002)
6-8°C	0.030***	0.040***			0.000	0.002
	(0.010)	(0.011)			(0.001)	(0.002)
8-10°C	0.004	0.008			-0.000	0.001
	(0.007)	(0.008)			(0.001)	(0.002)
10-12°C	0.017***	0.023***			-0.001	0.001
	(0.005)	(0.006)			(0.001)	(0.001)
12-14°C	0.012**	0.017***			-0.001	-0.000
	(0.005)	(0.005)			(0.001)	(0.001)
14-16°C	0.006	0.001			0.002*	0.002
	(0.004)	(0.005)			(0.001)	(0.001)
16-18°C	0.007**	0.009**			0.001*	0.000
	(0.003)	(0.004)			(0.001)	(0.001)
18-20°C	0.007**	0.009**			0.001*	0.001
	(0.003)	(0.004)			(0.001)	(0.001)
22-24°C	-0.001	0.005			0.000	0.000

	(0.003)	(0.003)			(0.001)	(0.001)
24-26°C	0.025***	0.028***			0.001	0.001
	(0.004)	(0.005)			(0.001)	(0.001)
26-28°C	0.025***	0.027***			0.001	0.002
	(0.005)	(0.006)			(0.001)	(0.001)
28-30°C	0.027***	0.031***			-0.001	-0.000
	(0.006)	(0.007)			(0.002)	(0.002)
30-32°C	0.022***	0.029***			-0.009***	-0.012***
	(0.008)	(0.010)			(0.003)	(0.004)
32-34°C	0.057***	0.072***			-0.008**	-0.018***
	(0.014)	(0.018)			(0.004)	(0.005)
>34°C	0.055***	0.071***			-0.010	-0.034***
	(0.019)	(0.021)			(0.007)	(0.008)
Temperature-21°C			0.004***	0.005***		
			(0.001)	(0.001)		
21°C-Temperature			0.002***	0.002***		
			(0.001)	(0.001)		
Wind speed	-0.002	-0.002	-0.002	-0.002	0.000	0.002***
	(0.002)	(0.002)	(0.002)	(0.002)	(0.000)	(0.001)
Precipitation	-0.001***	-0.002***	-0.001***	-0.002***	-0.000	0.000*
	(0.000)	(0.000)	(0.000)	(0.000)	(0.000)	(0.000)
Relative humidity	-0.006	-0.011*	-0.005	-0.010	-0.002*	-0.001
	(0.005)	(0.006)	(0.005)	(0.006)	(0.001)	(0.002)
Relative humidity^2	0.001*	0.001***	0.001	0.001***	0.000**	0.000
	(0.000)	(0.000)	(0.000)	(0.000)	(0.000)	(0.000)
lnprice	0.097***	0.006	0.097***	0.006	0.238***	0.226***
	(0.005)	(0.016)	(0.005)	(0.016)	(0.001)	(0.002)
Fixed effects						
County*week	Y	Y	Y	Y	Y	Y
State*year*month	Y	Y	Y	Y	Y	Y
N	1871472	1564506	1871472	1564506	25395497	19278726
R^2	0.187	0.196	0.187	0.196	0.453	0.383

Notes: Standard errors in parentheses are clustered to store level. Results weighted by the units sold per transaction. * p<0.1, ** p<0.05, *** p<0.01. The relative humidity multiplied by 10.

Supplementary Table 3 The effect of temperature on Energy Star air conditioner purchase in terms of CDDs and HDDs

	Major analysis		Current climate controlled		Lagged climate separated		Lagged climate summed	
	(1)	(2)	(3)	(4)	(5)	(6)	(7)	(8)
	Main	Price Outlier	Main	Price Outlier	Main	Price Outlier	Main	Price Outlier
CDD	0.004***	0.005***	0.004***	0.004***	0.005***	0.005***		
	(0.001)	(0.001)	(0.001)	(0.001)	(0.001)	(0.001)		
CDD, current week			-0.001	-0.001				
			(0.001)	(0.001)				
CDD, lagged 1 week					-0.000	-0.003***		
					(0.001)	(0.001)		
CDD, lagged 2 week					-0.001	0.001		
					(0.001)	(0.001)		
CDD, lagged 3 week					0.001*	0.003***		
					(0.001)	(0.001)		
CDD, 1 month							0.001***	0.001***
							(0.000)	(0.000)
HDD	0.002***	0.002***	0.001***	0.001**	0.002***	0.002***		
	(0.000)	(0.000)	(0.000)	(0.000)	(0.000)	(0.000)		
HDD, current week			-0.003***	-0.003***				
			(0.000)	(0.001)				
HDD, lagged 1 week					-0.000	-0.001***		
					(0.000)	(0.000)		
HDD, lagged 2 week					0.001***	0.000		
					(0.000)	(0.000)		
HDD, lagged 3 week					-0.000	-0.001**		
					(0.000)	(0.000)		
HDD, 1 month							0.001***	-0.000**

							(0.000)	(0.000)
Wind speed	-0.003*	-0.002	-0.001	-0.000	-0.002*	-0.002*		
	(0.002)	(0.002)	(0.001)	(0.002)	(0.001)	(0.001)		
Wind speed, current week			-0.002	-0.004**				
			(0.001)	(0.002)				
Wind speed, lagged 1 week					0.003***	0.007***		
					(0.001)	(0.001)		
Wind speed, lagged 2 week					0.001	0.004***		
					(0.001)	(0.001)		
Wind speed, lagged 3 week					-0.004***	-0.008***		
					(0.001)	(0.002)		
Wind speed, 1 month							-0.002	0.000
							(0.003)	(0.003)
Precipitation	-0.001***	-0.002***	-0.001***	-0.002***	-0.001***	-0.002***		
	(0.000)	(0.000)	(0.000)	(0.000)	(0.000)	(0.000)		
Precipitation, current week			-0.000	0.001				
			(0.000)	(0.000)				
Precipitation, lagged 1 week					0.001**	0.000		
					(0.000)	(0.000)		
Precipitation, lagged 2 week					-0.000	0.000		
					(0.000)	(0.000)		
Precipitation, lagged 3 week					-0.001**	-0.000		
					(0.000)	(0.000)		
Precipitation, 1 month							-0.001	-0.001*
							(0.001)	(0.001)
Relative humidity	-0.005	-0.011*	-0.007	-0.012*	-0.014***	-0.015**		

	(0.005)	(0.006)	(0.005)	(0.006)	(0.005)	(0.006)		
Relative humidity, current week			0.022***	0.028***				
			(0.005)	(0.007)				
Relative humidity, lagged 1 week					-0.011*	-0.008		
					(0.006)	(0.007)		
Relative humidity, lagged 2 week					0.035***	0.032***		
					(0.006)	(0.007)		
Relative humidity, lagged 3 week					0.016***	0.029***		
					(0.005)	(0.007)		
Relative humidity, 1 month							0.005	0.021*
							(0.010)	(0.013)
Relative humidity^2	0.001*	0.002***	0.001**	0.002***	0.001***	0.002***		
	(0.000)	(0.000)	(0.000)	(0.000)	(0.000)	(0.000)		
Relative humidity^2, current week			-0.002***	-0.003***				
			(0.000)	(0.001)				
Relative humidity^2, lagged 1 week					0.001**	0.001		
					(0.001)	(0.001)		
Relative humidity^2, lagged 2 week					-0.003***	-0.003***		
					(0.000)	(0.001)		
Relative humidity^2, lagged 3 week					-0.001**	-0.002***		
					(0.000)	(0.001)		
Relative humidity^2, 1 month							-0.000	-0.001
							(0.001)	(0.001)

lnprice	0.097***	0.006	0.097***	0.006	0.097***	0.005	0.097***	0.005
	(0.005)	(0.016)	(0.005)	(0.016)	(0.005)	(0.016)	(0.005)	(0.016)
Fixed effects								
County*week	Y	Y	Y	Y	Y	Y	Y	Y
State*year*month	Y	Y	Y	Y	Y	Y	Y	Y
N	1871472	1564506	1868326	1561994	1862247	1557351	1862247	1557351
R^2	0.187	0.196	0.188	0.196	0.188	0.196	0.187	0.196

Notes: Standard errors in parentheses are clustered to store level. Results weighted by the units sold per transaction. * p<0.1, ** p<0.05, *** p<0.01. The CDD, HDD, and relative humidity multiplied by 10.

Supplementary Table 4 Robustness check, 18°C as reference point

	(1)<br>Main	(2)<br>Price Outlier	(1)<br>Main	(2)<br>Price Outlier
<0°C	-0.007**	-0.009**		
	(0.003)	(0.004)		
0-2°C	-0.175***	-0.154***		
	(0.041)	(0.053)		
2-4°C	-0.029	-0.016		
	(0.019)	(0.021)		
4-6°C	-0.018	-0.009		
	(0.015)	(0.016)		
6-8°C	0.007	0.017		
	(0.010)	(0.011)		
8-10°C	0.023***	0.031***		
	(0.009)	(0.010)		
10-12°C	-0.003	-0.001		
	(0.006)	(0.007)		
12-14°C	0.011**	0.015***		
	(0.004)	(0.005)		
14-16°C	0.005	0.008**		
	(0.004)	(0.004)		
18-20°C	-0.001	-0.008**		

	(0.003)	(0.004)		
20-22°C	-0.000	0.000		
	(0.003)	(0.003)		
22-24°C	-0.008**	-0.003		
	(0.004)	(0.005)		
24-26°C	0.018***	0.019***		
	(0.005)	(0.006)		
26-28°C	0.018***	0.018***		
	(0.006)	(0.007)		
28-30°C	0.020***	0.022***		
	(0.006)	(0.007)		
30-32°C	0.015*	0.020*		
	(0.009)	(0.010)		
32-34°C	0.050***	0.063***		
	(0.014)	(0.018)		
>34°C	0.048**	0.063***		
	(0.019)	(0.021)		
CDD, 65 °F			0.002***	0.003***
			(0.000)	(0.001)
HDD, 65 °F			0.002***	0.003***
			(0.000)	(0.001)
Wind speed	-0.002	-0.002	-0.002	-0.002
	(0.002)	(0.002)	(0.002)	(0.002)
Precipitation	-0.001***	-0.002***	-0.001***	-0.002***
	(0.000)	(0.000)	(0.000)	(0.000)
Relative humidity	-0.006	-0.011*	-0.008	-0.012*
	(0.005)	(0.006)	(0.005)	(0.006)
Relative humidity^2	0.001*	0.001***	0.001**	0.002***
	(0.000)	(0.000)	(0.000)	(0.000)
lnprice	0.097***	0.006	0.097***	0.006
	(0.005)	(0.016)	(0.005)	(0.016)

Fixed effects				
County*week	Y	Y	Y	Y
State*year*month	Y	Y	Y	Y
N	1871472	1564506	1871472	1564506
R^2	0.187	0.196	0.187	0.196

Notes: Standard errors in parentheses are clustered to store level. Results weighted by the units sold per transaction. * p<0.1, ** p<0.05, *** p<0.01. The CDD, HDD, and relative humidity multiplied by 10.

Supplementary Table 5 Robustness check, nonlinear effect of meteorological indicators

	(1)<br>Main	(2)<br>Price Outlier	(3)<br>Main	(4)<br>Price Outlier
(Temperature-21°C)/10	0.050***	0.052***	0.050***	0.052***
	(0.013)	(0.016)	(0.013)	(0.016)
(Temperature-21°C) ^{2}/100	-0.005	-0.002	-0.005	-0.002
	(0.014)	(0.017)	(0.014)	(0.017)
(21°C-Temperature)/10	0.040***	0.027**	0.040***	0.027**
	(0.010)	(0.011)	(0.010)	(0.012)
(21°C-Temperature) ^{2}/100	-0.021***	-0.009	-0.021***	-0.009
	(0.007)	(0.008)	(0.007)	(0.008)
Wind speed	-0.002	-0.002	-0.003	-0.001
	(0.002)	(0.002)	(0.005)	(0.005)
Wind speed^2			0.000	-0.000
			(0.001)	(0.001)
Precipitation	-0.001***	-0.002***	-0.001**	-0.002***
	(0.000)	(0.000)	(0.001)	(0.001)
Precipitation^2			0.000	0.000
			(0.000)	(0.000)
Relative humidity	-0.005	-0.010	-0.005	-0.010
	(0.005)	(0.006)	(0.005)	(0.006)
Relative humidity^2	0.001	0.001***	0.001	0.001***
	(0.000)	(0.000)	(0.000)	(0.000)

lnprice	0.097***	0.006	0.097***	0.006
	(0.005)	(0.016)	(0.005)	(0.016)
Fixed effects				
County*week	Y	Y	Y	Y
State*year*month	Y	Y	Y	Y
N	1871472	1564506	1871472	1564506
R-sq	0.187	0.196	0.187	0.196

Notes: Standard errors in parentheses are clustered to store level. Results weighted by the units sold per transaction. * p<0.1, ** p<0.05, *** p<0.01. The relative humidity multiplied by 10.

Supplementary Table 6 Robustness check, different levels of clustering

	(1) County	(2) Zip Code, 3 digit	(3) Nielsen Designated Market Areas
CDD	0.004***	0.004***	0.004***
	(0.001)	(0.001)	(0.001)
HDD	0.002***	0.002***	0.002***
	(0.000)	(0.000)	(0.001)
Wind speed	-0.003	-0.003	-0.003
	(0.002)	(0.002)	(0.003)
Precipitation	-0.001***	-0.001***	-0.001***
	(0.000)	(0.000)	(0.000)
Relative humidity	-0.005	-0.005	-0.005
	(0.007)	(0.006)	(0.011)
Relative humidity^2	0.001	0.001	0.001
	(0.001)	(0.001)	(0.001)
lnprice	0.097***	0.097***	0.097***
	(0.008)	(0.008)	(0.015)
Fixed effects			
County*week	Y	Y	Y
State*year*month	Y	Y	Y

N	1871472	1871472	1871472
R^2	0.187	0.187	0.187

Notes: Results weighted by the units sold per transaction. * p<0.1, ** p<0.05, *** p<0.01. The CDD, HDD, and relative humidity multiplied by 10.

Supplementary Table 7 Robustness check, alternative combinations of fixed effects

	(1) EnergyStar	(2) EnergyStar	(3) EnergyStar	(4) EnergyStar	(5) EnergyStar
CDD	0.002***	0.003***	0.003***	0.003***	0.002***
	(0.001)	(0.001)	(0.001)	(0.001)	(0.001)
HDD	0.001***	0.002***	0.002***	0.001***	0.002***
	(0.000)	(0.000)	(0.000)	(0.000)	(0.000)
Wind speed	0.006***	0.007***	0.006***	0.006***	0.004**
	(0.002)	(0.002)	(0.002)	(0.002)	(0.002)
Precipitation	-0.002***	-0.002***	-0.002***	-0.002***	-0.002***
	(0.000)	(0.000)	(0.000)	(0.000)	(0.000)
Relative humidity	-0.014***	-0.015***	-0.015***	-0.013***	-0.010**
	(0.005)	(0.005)	(0.005)	(0.005)	(0.004)
Relative humidity^2	0.002***	0.002***	0.002***	0.002***	0.001***
	(0.000)	(0.000)	(0.000)	(0.000)	(0.000)
lnprice	0.099***	0.100***	0.100***	0.100***	0.096***
	(0.005)	(0.005)	(0.005)	(0.005)	(0.005)
Fixed effects					
County*week		Y	Y	Y	Y
County*month	Y				
State*year					Y
State*month				Y	
State	Y	Y	Y		
Year	Y	Y	Y	Y	
Month			Y		Y
Week	Y				

N	1879080	1871984	1871984	1871982	1871981
R^2	0.128	0.144	0.144	0.146	0.169

Notes: Standard errors in parentheses are clustered to store level. Results weighted by the units sold per transaction. * p<0.1, ** p<0.05, *** p<0.01. The CDD, HDD, and relative humidity multiplied by 10.

Supplementary Table 8 The effect of weather on air conditioner price

	(1) Main	(2) Price Outlier
EnergyStar	0.285***	0.203***
	(0.048)	(0.053)
CDD	-0.001	-0.004***
	(0.001)	(0.001)
EnergyStar*CDD	-0.010***	0.006***
	(0.001)	(0.002)
HDD	0.002***	0.001***
	(0.001)	(0.000)
EnergyStar*HDD	-0.008***	-0.003***
	(0.001)	(0.001)
Wind speed	0.004	0.012***
	(0.002)	(0.003)
EnergyStar*Wind speed	-0.016***	-0.039***
	(0.005)	(0.007)
Precipitation	-0.000	-0.000
	(0.000)	(0.001)
EnergyStar*Precipitation	0.003***	0.001
	(0.001)	(0.001)
Relative humidity	-0.036***	-0.024***
	(0.009)	(0.008)
EnergyStar*Relative humidity	-0.016	-0.012
	(0.014)	(0.015)
Relative humidity^2	0.003***	0.003***

	(0.001)	(0.001)
EnergyStar*Relative humidity^2	0.001	-0.000
	(0.001)	(0.001)
Fixed effects		
County*week	Y	Y
State*year*month	Y	Y
N	1871472	1564506
R^2	0.280	0.276

Notes: Standard errors in parentheses are clustered to store level. Results weighted by the units sold per transaction. * p<0.1, ** p<0.05, *** p<0.01. The CDD, HDD, and relative humidity multiplied by 10.

Supplementary Table 9 The effect of temperature on Energy Star telephone purchase in terms of CDDs and HDDs

	Major analysis		Current climate controlled		Lagged climate separated		Lagged climate summed	
	(1)	(2)	(3)	(4)	(5)	(6)	(7)	(8)
	Main	Price Outlier	Main	Price Outlier	Main	Price Outlier	Main	Price Outlier
CDD	-0.000*	-0.000*	0.000	-0.000	-0.000	0.000		
	(0.000)	(0.000)	(0.000)	(0.000)	(0.000)	(0.000)		
CDD, current week			-0.001***	-0.001***				
			(0.000)	(0.000)				
CDD, lagged 1 week					-0.000	-0.000		
					(0.000)	(0.000)		
CDD, lagged 2 week					0.000**	0.000		
					(0.000)	(0.000)		
CDD, lagged 3 week					-0.001***	-0.001***		
					(0.000)	(0.000)		
CDD, 1 month							-0.000	-0.000
							(0.000)	(0.000)
HDD	0.000**	0.000***	0.000**	0.000***	0.000*	0.000***		
	(0.000)	(0.000)	(0.000)	(0.000)	(0.000)	(0.000)		

HDD, current week			0.000	-0.000				
			(0.000)	(0.000)				
HDD, lagged 1 week					0.000*	0.000***		
					(0.000)	(0.000)		
HDD, lagged 2 week					0.000	-0.000***		
					(0.000)	(0.000)		
HDD, lagged 3 week					0.000***	0.001***		
					(0.000)	(0.000)		
HDD, 1 month							0.000***	0.000***
							(0.000)	(0.000)
Wind speed	-0.000	0.002***	0.000	0.002***	-0.000	0.001***		
	(0.000)	(0.001)	(0.000)	(0.000)	(0.000)	(0.000)		
Wind speed, current week			-0.000	0.000				
			(0.000)	(0.000)				
Wind speed, lagged 1 week					0.001***	0.001***		
					(0.000)	(0.000)		
Wind speed, lagged 2 week					0.001**	0.001***		
					(0.000)	(0.000)		
Wind speed, lagged 3 week					-0.000	-0.000		
					(0.000)	(0.000)		
Wind speed, 1 month							0.001	0.003***
							(0.001)	(0.001)
Precipitation	-0.000	0.000*	-0.000*	0.000*	-0.000	0.000**		
	(0.000)	(0.000)	(0.000)	(0.000)	(0.000)	(0.000)		
Precipitation, current week			0.000	0.000**				
			(0.000)	(0.000)				
Precipitation, lagged 1 week					0.000	0.000***		
					(0.000)	(0.000)		

Precipitation, lagged 2 week					-0.000**	-0.000**		
					(0.000)	(0.000)		
Precipitation, lagged 3 week					-0.000***	-0.000**		
					(0.000)	(0.000)		
Precipitation, 1 month							-0.000*	0.000
							(0.000)	(0.000)
Relative humidity	-0.002	-0.000	-0.003**	-0.001	-0.002**	-0.001		
	(0.001)	(0.002)	(0.001)	(0.002)	(0.001)	(0.002)		
Relative humidity, current week			-0.002*	0.001				
			(0.001)	(0.002)				
Relative humidity, lagged 1 week					0.006***	0.009***		
					(0.001)	(0.001)		
Relative humidity, lagged 2 week					-0.004***	-0.004***		
					(0.001)	(0.001)		
Relative humidity, lagged 3 week					-0.003**	-0.004**		
					(0.001)	(0.001)		
Relative humidity, 1 month							-0.002	0.003
							(0.004)	(0.005)
Relative humidity^2	0.000**	0.000	0.000***	0.000	0.000***	0.000		
	(0.000)	(0.000)	(0.000)	(0.000)	(0.000)	(0.000)		
Relative humidity^2, current week			0.000	-0.000				
			(0.000)	(0.000)				
Relative humidity^2, lagged 1 week					-0.000***	-0.001***		
					(0.000)	(0.000)		

Relative humidity^2, lagged 2 week					0.000***	0.000***		
					(0.000)	(0.000)		
Relative humidity^2, lagged 3 week					0.000**	0.000*		
					(0.000)	(0.000)		
Relative humidity^2, 1 month							0.000	-0.000
							(0.000)	(0.000)
lnprice	0.238***	0.226***	0.238***	0.226***	0.238***	0.226***	0.238***	0.226***
	(0.001)	(0.002)	(0.001)	(0.002)	(0.001)	(0.002)	(0.001)	(0.002)
Fixed effects								
County*week	Y	Y	Y	Y	Y	Y	Y	Y
State*year*month	Y	Y	Y	Y	Y	Y	Y	Y
N	25395497	19278726	25226932	19157396	24940700	18951915	24940700	18951915
R^2	0.453	0.383	0.453	0.383	0.453	0.383	0.453	0.383

Notes: Standard errors in parentheses are clustered to store level. Results weighted by the units sold per transaction. * p<0.1, ** p<0.05, *** p<0.01. The CDD, HDD, and relative humidity multiplied by 10.

Supplementary Table 10 Heterogeneous effect, climate and electricity price

	(1)<br>Electricity price	(2)<br>CDD	(3)<br>HDD
CDD	0.000	0.008***	0.001
	(0.001)	(0.002)	(0.001)
CDD*group 2	0.002*	-0.002	0.008***
	(0.001)	(0.002)	(0.001)
CDD*group 3	0.008***	-0.006***	0.001
	(0.001)	(0.002)	(0.002)
HDD	-0.001**	0.002***	0.001**
	(0.001)	(0.001)	(0.001)
HDD*group 2	0.003***	0.001	0.001

	(0.001)	(0.001)	(0.001)
HDD*group 3	0.005***	-0.001	0.001
	(0.001)	(0.001)	(0.001)
Wind speed	-0.001	-0.006**	0.001
	(0.002)	(0.003)	(0.002)
Wind speed*group 2	0.004	0.006	-0.003
	(0.003)	(0.004)	(0.003)
Wind speed*group 3	-0.005	0.008**	-0.004
	(0.003)	(0.003)	(0.003)
Precipitation	0.000	-0.001**	0.000
	(0.000)	(0.001)	(0.000)
Precipitation*group 2	-0.001**	-0.001	-0.002***
	(0.001)	(0.001)	(0.001)
Precipitation*group 3	-0.002***	0.002**	-0.001
	(0.001)	(0.001)	(0.001)
Relative humidity	-0.018*	-0.002	-0.002
	(0.009)	(0.011)	(0.006)
Relative humidity*group 2	0.008	-0.014	-0.029**
	(0.014)	(0.014)	(0.014)
Relative humidity*group 3	0.031***	-0.005	-0.003
	(0.012)	(0.013)	(0.012)
Relative humidity^2	0.001*	-0.000	0.000
	(0.001)	(0.001)	(0.000)
Relative humidity^2*group 2	-0.001	0.002**	0.003***
	(0.001)	(0.001)	(0.001)
Relative humidity^2*group 3	-0.002*	0.001	-0.000
	(0.001)	(0.001)	(0.001)
lnprice	0.097***	0.097***	0.097***
	(0.005)	(0.005)	(0.005)
Fixed effects			
County*week	Y	Y	Y

State*year*month	Y	Y	Y
N	1871472	1871326	1871326
R^2	0.187	0.187	0.187

Notes: Standard errors in parentheses are clustered to store level. Results weighted by the units sold per transaction. * p<0.1, ** p<0.05, *** p<0.01. The CDD, HDD, and relative humidity multiplied by 10.

Supplementary Table 11 Heterogeneous effect, socio-economic characteristics

	(1)<br>Income	(2)<br>Education	(3)<br>Ethnic	(4)<br>Age
CDD	0.002	0.002*	0.004***	0.003**
	(0.001)	(0.001)	(0.001)	(0.001)
CDD*group 2	0.001	0.001	-0.002	0.000
	(0.002)	(0.002)	(0.002)	(0.002)
CDD*group 3	0.003*	0.003	0.001	0.003
	(0.002)	(0.002)	(0.002)	(0.002)
HDD	-0.001	-0.001	0.003***	0.000
	(0.001)	(0.001)	(0.001)	(0.001)
HDD*group 2	0.003***	0.002**	-0.001	0.001
	(0.001)	(0.001)	(0.001)	(0.001)
HDD*group 3	0.003***	0.005***	-0.003***	0.002
	(0.001)	(0.001)	(0.001)	(0.001)
Wind speed	0.002	-0.003	0.005*	0.002
	(0.003)	(0.003)	(0.003)	(0.003)
Wind speed*group 2	-0.002	0.006*	-0.003	-0.001
	(0.004)	(0.004)	(0.004)	(0.004)
Wind speed*group 3	-0.002	0.002	-0.013***	-0.004
	(0.004)	(0.004)	(0.004)	(0.004)
Precipitation	-0.001	0.000	-0.001	-0.000
	(0.001)	(0.001)	(0.001)	(0.001)

Precipitation*group 2	0.001	-0.002*	-0.001	-0.001
	(0.001)	(0.001)	(0.001)	(0.001)
Precipitation*group 3	-0.002**	-0.003**	-0.002**	-0.003**
	(0.001)	(0.001)	(0.001)	(0.001)
Relative humidity	0.013	0.002	-0.003	-0.001
	(0.012)	(0.011)	(0.008)	(0.008)
Relative humidity*group 2	-0.017	-0.001	0.009	-0.008
	(0.017)	(0.017)	(0.012)	(0.013)
Relative humidity*group 3	-0.040**	-0.026	-0.028*	-0.020
	(0.016)	(0.017)	(0.014)	(0.018)
Relative humidity^2	-0.001	0.000	0.001	0.000
	(0.001)	(0.001)	(0.001)	(0.001)
Relative humidity^2*group 2	0.002	0.001	-0.001	0.000
	(0.001)	(0.001)	(0.001)	(0.001)
Relative humidity^2*group 3	0.003***	0.002	0.002	0.002
	(0.001)	(0.001)	(0.001)	(0.001)
lnprice	0.080***	0.080***	0.080***	0.080***
	(0.005)	(0.005)	(0.005)	(0.005)
Fixed effects				
County*week	Y	Y	Y	Y
State*year*month	Y	Y	Y	Y
N	1474685	1474685	1474685	1474685
R^2	0.208	0.208	0.208	0.208

Notes: Standard errors in parentheses are clustered to store level. Results weighted by the units sold per transaction. * p<0.1, ** p<0.05, *** p<0.01. The CDD, HDD, and relative humidity multiplied by 10.

Supplementary Table 12 Heterogeneous effect, housing characteristics

	(1) Rooms	(2) Owner	(3) Fuel
CDD	0.003**	0.003**	0.006***
	(0.001)	(0.001)	(0.002)
CDD*group 2	0.002	0.000	-0.003*

	(0.002)	(0.002)	(0.002)
CDD*group 3	0.002	0.004*	-0.005***
	(0.002)	(0.002)	(0.002)
HDD	0.002*	0.003***	0.002**
	(0.001)	(0.001)	(0.001)
HDD*group 2	-0.001	-0.001	-0.000
	(0.001)	(0.001)	(0.001)
HDD*group 3	0.000	-0.003**	-0.001
	(0.001)	(0.001)	(0.001)
Wind speed	0.001	0.003	-0.001
	(0.002)	(0.003)	(0.003)
Wind speed*group 2	-0.002	-0.001	0.004
	(0.004)	(0.003)	(0.004)
Wind speed*group 3	-0.004	-0.009**	0.000
	(0.004)	(0.004)	(0.003)
Precipitation	-0.000	0.000	-0.002***
	(0.001)	(0.001)	(0.001)
Precipitation*group 2	-0.003**	-0.003***	-0.000
	(0.001)	(0.001)	(0.001)
Precipitation*group 3	-0.001	-0.002*	0.003***
	(0.001)	(0.001)	(0.001)
Relative humidity	0.005	0.011	-0.036***
	(0.007)	(0.007)	(0.013)
Relative humidity*group 2	-0.036**	-0.027**	0.042***
	(0.015)	(0.012)	(0.015)
Relative humidity*group 3	-0.042**	-0.057***	0.043***
	(0.017)	(0.014)	(0.017)
Relative humidity^2	-0.000	-0.001	0.003***
	(0.001)	(0.001)	(0.001)
Relative humidity^2*group 2	0.003**	0.002**	-0.004***
	(0.001)	(0.001)	(0.001)
Relative humidity^2*group 3	0.003**	0.005***	-0.004***

	(0.001)	(0.001)	(0.001)
lnprice	0.081***	0.081***	0.081***
	(0.005)	(0.005)	(0.005)
Fixed effects			
County*week	Y	Y	Y
State*year*month	Y	Y	Y
N	1474685	1474685	1474685
R^2	0.208	0.208	0.208

Notes: Standard errors in parentheses are clustered to store level. Results weighted by the units sold per transaction. * p<0.1, ** p<0.05, *** p<0.01. The CDD, HDD, and relative humidity multiplied by 10.

Supplementary Table 13 Heterogeneous effect, climate attitude

	(1) Believe climate change happening	(2) Believe climate change harm US	(3) Worry about climate change	(4) Support renewable energy standards	(5) Support regulation of CO_2	(6) Support Democratic party
CDD	0.003***	0.001	0.003***	0.003***	0.002**	0.002**
	(0.001)	(0.001)	(0.001)	(0.001)	(0.001)	(0.001)
CDD*group 2	0.002	0.003**	0.000	0.002	0.001	0.002*
	(0.001)	(0.001)	(0.001)	(0.001)	(0.001)	(0.001)
CDD*group 3	0.002	0.004***	0.004***	0.002	0.004***	0.003**
	(0.001)	(0.001)	(0.001)	(0.001)	(0.001)	(0.001)
HDD	-0.000	-0.000	-0.001	-0.001	-0.001	0.001
	(0.001)	(0.001)	(0.001)	(0.001)	(0.001)	(0.001)
HDD*group 2	0.002***	0.002***	0.003***	0.003***	0.002***	0.002***
	(0.001)	(0.001)	(0.001)	(0.001)	(0.001)	(0.001)
HDD*group 3	0.004***	0.004***	0.004***	0.004***	0.004***	0.001
	(0.001)	(0.001)	(0.001)	(0.001)	(0.001)	(0.001)
Wind speed	-0.000	0.000	-0.001	-0.001	0.001	-0.001
	(0.002)	(0.002)	(0.002)	(0.002)	(0.002)	(0.002)

Wind speed*group 2	-0.005	-0.003	0.002	-0.007**	-0.004	-0.002
	(0.003)	(0.003)	(0.003)	(0.003)	(0.003)	(0.003)
Wind speed*group 3	-0.001	-0.004	-0.004	0.002	-0.005	-0.002
	(0.003)	(0.003)	(0.003)	(0.003)	(0.003)	(0.003)
Precipitation	-0.001*	-0.000	-0.001	-0.001	-0.001	-0.001*
	(0.000)	(0.000)	(0.000)	(0.000)	(0.000)	(0.000)
Precipitation*group 2	-0.001	-0.001	-0.000	0.000	-0.000	0.000
	(0.001)	(0.001)	(0.001)	(0.001)	(0.001)	(0.001)
Precipitation*group 3	-0.001	-0.002**	-0.001	-0.001*	-0.001*	-0.001
	(0.001)	(0.001)	(0.001)	(0.001)	(0.001)	(0.001)
Relative humidity	-0.039***	-0.019*	-0.026**	-0.023***	-0.018*	-0.008
	(0.010)	(0.011)	(0.011)	(0.009)	(0.010)	(0.009)
Relative humidity*group 2	0.044***	0.007	0.014	0.028**	0.015	-0.006
	(0.013)	(0.014)	(0.014)	(0.012)	(0.012)	(0.013)
Relative humidity*group 3	0.039***	0.021	0.031**	0.024**	0.025**	0.016
	(0.012)	(0.013)	(0.013)	(0.011)	(0.012)	(0.012)
Relative humidity^2	0.003***	0.001*	0.002**	0.002***	0.002**	0.001
	(0.001)	(0.001)	(0.001)	(0.001)	(0.001)	(0.001)
Relative humidity^2*group 2	-0.003***	-0.001	-0.001	-0.002**	-0.001	0.001
	(0.001)	(0.001)	(0.001)	(0.001)	(0.001)	(0.001)
Relative humidity^2*group 3	-0.003***	-0.001	-0.002*	-0.002*	-0.002	-0.001
	(0.001)	(0.001)	(0.001)	(0.001)	(0.001)	(0.001)
lnprice	0.097***	0.097***	0.097***	0.097***	0.097***	0.097***
	(0.005)	(0.005)	(0.005)	(0.005)	(0.005)	(0.005)
Fixed effects						
County*week	Y	Y	Y	Y	Y	Y
State*year*month	Y	Y	Y	Y	Y	Y
N	1870598	1870598	1870598	1870598	1870598	1869917
R^2	0.187	0.187	0.187	0.187	0.187	0.187

Notes: Standard errors in parentheses are clustered to store level. Results weighted by the units sold per transaction. * p<0.1, ** p<0.05, *** p<0.01. The CDD, HDD, and relative humidity multiplied by 10.
