# Supplementary material for: The weather affects air conditioner purchases to fill the energy efficiency gap
Source: Nat Commun. 2022 Oct 1;13:5772. doi: 10.1038/s41467-022-33531-2 (PMC9526731; doi:10.1038/s41467-022-33531-2)
Supplement: Supplementary file 3 — Reporting Summary [file 41467_2022_33531_MOESM3_ESM.pdf]

nature portfolio

Corresponding author(s): Pengfei Liu, Yueming Qiu

Last updated by author(s): Sep 15, 2022

Reporting Summary

Nature Portfolio wishes to improve the reproducibility of the work that we publish. This form provides structure for consistency and transparency in reporting. For further information on Nature Portfolio policies, see our Editorial Policies and the Editorial Policy Checklist.

Statistics

For all statistical analyses, confirm that the following items are present in the figure legend, table legend, main text, or Methods section.

n/a	Confirmed	
☐	☒	The exact sample size (*n*) for each experimental group/condition, given as a discrete number and unit of measurement
☐	☒	A statement on whether measurements were taken from distinct samples or whether the same sample was measured repeatedly
☐	☒	The statistical test(s) used AND whether they are one- or two-sided<br>*Only common tests should be described solely by name; describe more complex techniques in the Methods section.*
☐	☒	A description of all covariates tested
☐	☒	A description of any assumptions or corrections, such as tests of normality and adjustment for multiple comparisons
☐	☒	A full description of the statistical parameters including central tendency (e.g. means) or other basic estimates (e.g. regression coefficient) AND variation (e.g. standard deviation) or associated estimates of uncertainty (e.g. confidence intervals)
☐	☒	For null hypothesis testing, the test statistic (e.g. *F*, *t*, *r*) with confidence intervals, effect sizes, degrees of freedom and *P* value noted<br>*Give P values as exact values whenever suitable.*
☒	☐	For Bayesian analysis, information on the choice of priors and Markov chain Monte Carlo settings
☒	☐	For hierarchical and complex designs, identification of the appropriate level for tests and full reporting of outcomes
☒	☐	Estimates of effect sizes (e.g. Cohen's *d*, Pearson's *r*), indicating how they were calculated

Our web collection on statistics for biologists contains articles on many of the points above.

Software and code

Policy information about availability of computer code

Data collection	The GSOD data are accessed using GSODR package in R studio (based on R 4.0.2). The code for data download can be found at https://github.com/hepannju/Does-the-weather-change-the-energy-efficiency-gap. Other data are downloaded from multiple websites (see the details in data availability statement) in the forms as they are and processed in Stata 16.0.
Data analysis	The major data processing and all the regression analysis are conducted in Stata 16.0. The download of GSOD data and figure production is conducted in R studio (based on R 4.0.2). The code for data analysis can be found at https://github.com/hepannju/Does-the-weather-change-the-energy-efficiency-gap.

For manuscripts utilizing custom algorithms or software that are central to the research but not yet described in published literature, software must be made available to editors and reviewers. We strongly encourage code deposition in a community repository (e.g. GitHub). See the Nature Portfolio guidelines for submitting code & software for further information.

Data

Policy information about availability of data

All manuscripts must include a data availability statement. This statement should provide the following information, where applicable:

- Accession codes, unique identifiers, or web links for publicly available datasets
- A description of any restrictions on data availability
- For clinical datasets or third party data, please ensure that the statement adheres to our policy

The GSOD data are accessed using GSODR package in R and can also be retrieved from https://www1.ncdc.noaa.gov/pub/data/gsod/. The Retail Scanner data is provided by Nielsen Company restricted by non-disclosure terms of use but can be purchased from Nielsen. The metrics of climate attitude are available in the supplementary material of the study "Geographic variation in opinions on climate change at state and local scales in the USA"(Howe et al., 2015). The annual state-level electricity price is obtained from the U.S. Energy Information Administration website at https://www.eia.gov/electricity/data/state/. The county-level socioeconomic and demographic characteristics are available in the American Community Survey from https://www.census.gov/geographies/mapping-files/time-series/geo/tiger-data.html. The support of the democratic party in county presidential election returns of 2000-2020 comes from at https://dataverse.harvard.edu/dataset.xhtml?persistentId=doi:10.7910/DVN/VOQCHQ

Human research participants

Policy information about studies involving human research participants and Sex and Gender in Research.

Reporting on sex and gender	Sex and gender related analyses are not included in this study.
Population characteristics	The study is based on second-hand purchase data provided by Nielsen company, which does not include individual-level population characteristics information.
Recruitment	No recruitment is conducted for this study.
Ethics oversight	he study is based on second-hand purchase data provided by Nielsen company. We did not conduct primary data collection involves human subjects. Ethics oversight is not applicable.

Note that full information on the approval of the study protocol must also be provided in the manuscript.

Field-specific reporting

Please select the one below that is the best fit for your research. If you are not sure, read the appropriate sections before making your selection.

☐ Life sciences ☒ Behavioural & social sciences ☐ Ecological, evolutionary & environmental sciences

For a reference copy of the document with all sections, see nature.com/documents/nr-reporting-summary-flat.pdf

Behavioural & social sciences study design

All studies must disclose on these points even when the disclosure is negative.

Study description	Investigate the relationship of weather and energy efficiency gap based on air conditioner purchase. The study is quantitative.
Research sample	General purchase outcomes provided by Nielsen Company, used for its representativeness. The retail scanner dataset contains information from approximately 30,000-50,000 individual stores from approximately 90 retail chains (these numbers vary by year). It is the most inclusive, nationwide, and longitude dataset for air conditioner transactions, which fits the data demand of this research for variation of weather and geographic/socioeconomic features, as well as details of the air conditioner transactions.
Sampling strategy	General purchase outcomes provided by Nielsen Company. Sampling strategy is controlled by Nielsen. The sampling is not based on a randomization strategy but depend more on the accessibility of the retail chains. However, given its broad coverage across the country and the large amount of stores/transactions, it provides decent reflection on consumer behaviour.
Data collection	Individual air conditioner data collected by Nielsen at the store level. Each individual store reports weekly data for every UPC code that had any sales volume during the week. Information captured for each UPC code includes: week ending date, units sold, weighted average unit price, etc. The resulted data that we access thus contains weekly summary of the these transactions including the UPC codes and description of the air conditioner models, total numbers of the transactions, the average of transaction price, 3-digit county code of the stores, etc.
Timing	The data collection started in 2006 and is updated every year. There is no gap between collection periods, sate, and dates. At the time of analysis, the data are available for 2006-2019 and thus we included the data of this period in the analysis.

Data exclusions	No data is excluded.
Non-participation	There is no non-participation at the consumer level as once a store agrees to participate in the data collection, the purchase data is recorded automatically.
Randomization	Randomization is not used in this study.

Reporting for specific materials, systems and methods

We require information from authors about some types of materials, experimental systems and methods used in many studies. Here, indicate whether each material, system or method listed is relevant to your study. If you are not sure if a list item applies to your research, read the appropriate section before selecting a response.

Materials & experimental systems

n/a	Involved in the study
☒	☐ Antibodies
☒	☐ Eukaryotic cell lines
☒	☐ Palaeontology and archaeology
☒	☐ Animals and other organisms
☒	☐ Clinical data
☒	☐ Dual use research of concern

Methods

n/a	Involved in the study
☒	☐ ChIP-seq
☒	☐ Flow cytometry
☒	☐ MRI-based neuroimaging
